# Successful insertion and expression of a tetracycline transactivator in *Anopheles stephensi* associated with increased egg production and decreased hatching rate

**DOI:** 10.1186/s13071-025-07003-7

**Published:** 2025-10-28

**Authors:** Ehud Inbar, Ishaan Samantray, Robert T. Alford, Robert A. Harrell, Grace Jennings, Tales V. Pascini, Tint T. Wai, Franck Dumetz, Abraham G. Eappen, Stephen L. Hoffman, Peter F. Billingsley

**Affiliations:** 1 Sanaria Inc. Rockville, Rockville, MD 20850 USA; 2 Insect Transformation Facility, Institute for Bioscience and Biotechnology Research, University of Maryland, Rockville, MD 20850 USA; 3 The Vital Narrative, Frederick, MD 21701 USA; 4 Institute for Genome Sciences, University of Maryland, Baltimore, MD USA

**Keywords:** Anopheles, Driver line, Tetracycline, rtTA

## Abstract

**Background:**

Sanaria^®^ has pioneered production of aseptic, purified, vialed cryopreserved *Plasmodium falciparum* (Pf) sporozoites (SPZ) as vaccines and for controlled human malaria infections. More than 3500 individuals have received more than 9700 injections of PfSPZ worldwide. The PfSPZ are manufactured in aseptically reared female *Anopheles stephensi* mosquitoes. Since PfSPZ vaccines are intended primarily for some of the most disadvantaged people in the world, keeping costs low is imperative. One approach to reducing the cost of goods is to eliminate male mosquitoes from the production process, thereby doubling the numbers of PfSPZ-producing mosquitoes per unit space. We intend to do this by creating *An. stephensi* with a male-lethal allele controlled by the tetracycline conditional gene expression system.

**Methods:**

Herein, we report the first step in this process, the creation of a driver line that expresses the reverse tetracycline transactivator (rtTA).

**Results:**

After suboptimal results using the *bZip* early embryonic promoter, we produced three mosquito driver lines that expressed rtTA from three different genomic loci under the early embryonic *vasa* promoter. Expressing the rtTA under the *vasa* promoter significantly increased rtTA mRNA levels compared with under *bZip*. We were unable to achieve homozygosity in two of these lines even after 26 generations. In a third line we observed seven distinct insertions, six of which, including one located in an intron of a protein-coding gene, were homozygous. This line achieved homozygosity after being passed through seven generations, suggesting that the insertions did not disrupt the function of any crucial genomic locus. The levels of rtTA mRNA expression in the homozygous-viable line were higher than those in the other two lines, reinforcing the idea that the inability to reach homozygosity was not due to rtTA expression levels but rather the position of the insertion. The homozygous-viable line produced ~18% more eggs per female, and a hatching rate of larvae from eggs was 39% lower than that of wild-type *An. stephensi*. The next step will be to cross the driver line with an effector line containing a male-linked lethal gene regulated by the tetracycline responsive element (TRE).

**Graphical Abstract:**

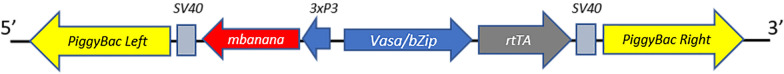

**Supplementary Information:**

The online version contains supplementary material available at 10.1186/s13071-025-07003-7.

## Background

Vector-borne diseases have a devastating impact worldwide, transmitting viruses, bacteria, and parasites, which account for 17% of all infectious diseases with more than 700,000 deaths annually [1]. Malaria is caused by *Plasmodium* parasites, which are transmitted by anopheline mosquitoes. In 2023, malaria caused 263 million illnesses and 597,000 deaths, mainly in young children [1]. Dengue virus is transmitted by *Aedes* mosquitoes, which poses a risk for roughly 4 billion people in 132 countries with annual mortality rate of 40,000 individuals [1] 

Sanaria’s *Plasmodium falciparum* (Pf) sporozoite (SPZ) products are composed of live, aseptic, purified cryopreserved PfSPZ [2]. More than 3500 individuals have received more than 9700 doses of PfSPZ products in 44 clinical trials in 14 countries in North America, Europe, Africa, and Asia [3–31]. Sanaria^®^ PfSPZ vaccines are safe and well tolerated and have achieved 100% efficacy against heterologous-controlled human malaria infection at 12 weeks after last dose of vaccine [29], sustained protection for at least 2 years against field-transmitted malaria in Africa, including 57% protection against Pf infection for 2 years with no boosting in Mali [32], and protection for at least 6 months against highly variant Pf parasites in Papua (Indonesian West New Guinea) (unpublished). Sanaria is currently clinically testing its third generation vaccine, genetically attenuated PfSPZ-LARC2 vaccine [33]. Recent research conducted in the Netherlands has demonstrated that a genetically attenuated vaccine strain with just a single deletion of the* mei2* gene (GA2) exhibits a vaccine efficacy of approximately 90% and is well tolerated [34, 35]. Given that PfSPZ-LARC2 is a double knockout vaccine strain, with deletions in both *mei2* and *LINUP*, we anticipate a similar or potentially greater protective efficacy, possibly achievable with just one dose.

All PfSPZ products are currently produced in aseptic *Anopheles stephensi* mosquitoes. Major drivers of the cost of the vaccine are the incubator space and the personnel required for production and management of aseptic *An. stephensi*. Only female mosquitoes carry the PfSPZ used in PfSPZ vaccines. If males could be eliminated from the manufacturing process, we would be able to produce double the numbers of PfSPZ in the same space and with the same personnel, thereby significantly reducing the cost of goods.

The first step in creating “female only” *An. stephensi* mosquitoes was to generate a driver line for conditional expression. To do this we used a tetracycline-controlled transcription mechanism, one that is central to tetracycline resistance of gram-negative bacteria [36], and has been adapted to conditionally express genes in different eukaryotic systems, including mammals and insects [37–41]. In this system, a gene of interest is regulated by the tetracycline response element (TRE), which consists of multiple units of the tetracycline operator (TetO). Transcription is initiated only when the tetracycline transactivator (tTA), a DNA-binding protein, binds to the TRE [37, 41, 42]. There are two main mechanistic adaptations of this system, Tet-On and Tet-Off. In the Tet-Off system, the tTA is bound to tetracycline or its derivative doxycycline to prevent binding of the tTA to the TRE; when the drug is removed, the tTA binds to the TRE to initiate transcription of the gene of interest. Conversely, in the Tet-On system, the tTA is modified to a reverse tetracycline transactivator (rtTA), which binds to the TRE in the presence of doxycycline to initiate transcription. When the drug is removed, the rtTA is released from the TRE and transcription stops [37, 41, 42].

The Tet system has been used in vector control approaches to decrease populations of various disease-transmitting vectors, using release of insects carrying a dominant lethal gene (RIDL). RIDL is an improved version of the classical release of sterile insect technique (SIT) [43]. Male *Aedes aegypti* mosquitoes carrying lethal genes are released to mate with wild-type females and transmit the lethal genes to future generations. In the absence of tetracycline or doxycycline in the field, lethal genes are induced, and the mosquitoes are killed, usually at the early larval stage [39, 40, 44–48]. Currently, there is an extensive effort to use RIDL to reduce populations of *Ae. aegypti* to fight the spread of arboviruses, including Zika, yellow fever, and dengue [46, 49, 50].

Our plan is to use the tetracycline conditional expression system to drive expression of a male lethal gene, thereby creating “female-only” aseptic mosquitoes for PfSPZ production. Here we report our successful generation of a homozygous driver line of mosquitoes expressing rtTA (Tet-On) to be used to drive expression of a male lethal gene.

## Methods

### Mosquito rearing

*Anopheles stephensi* SDA500 were reared at 28 °C, 75% humidity and 12:12 h light:dark cycle with 30-min dawn and dusk periods at the Insect Transformation Facility (ITF) of the University of Maryland’s Institute for Bioscience and Biotechnology Research (IBBR) in Rockville, MD, USA. Using a water-heated glass feeder, 7–8-day old adult females were blood-fed, using Parafilm as the feeding membrane. Eggs were collected 4 days post-feeding and were allowed to hatch in 1 L of water, supplemented with 15 mL of baker’s yeast (Fleishmann’s USA), derived from a 20% slurry in water. Approximately 150 larvae were distributed into pans, each containing one TetraMin tropical tablet (Tetra USA), 1 week post-feeding. Subsequently, 11 days post-hatching, transgenic lines were sorted (if needed), and an additional TetraMin tablet was introduced. Pupae were collected 12–14 days post-feeding and transferred into cups of water, which were then placed in adult cages. The pupae cups were removed 18 days post-feeding, and adults were provided with cotton soaked in 15% sucrose solution.

### Construction of driver lines: construction of vasa-rtTA in piggyBac vector and embryo injections

The reverse tetracycline transactivator (rtTA) gene, fused to VP16 transcription activation domain, was synthesized by GeneScript company (Piscataway NJ, USA). The *vasa* promoter was amplified from *An. stephensi* genomic DNA. We assembled *vasa*-rtTA-SV40 using NEBuilder^®^ HiFi DNA Assembly Cloning Kit (NEB USA). The entire construct was ligated into the *piggyBac* transposon vector containing mBanana gene under the *3xP3* promoter [51] for easy identification of transgenic *An*. *stephensi*. The injections were carried out as described previously [52, 53]. Briefly, preblastodermal embryos were injected, 40–60 min post-embryo collection, with an injection mix consisting of 150 ng/µL of *piggyBac* vector and 193 ng/µL of *piggyBac* transposase mRNA in halocarbon oil, as described [53].

### DNA work, Splinkerette mapping, and RNA work

DNA was extracted from individual adult mosquitoes using DNeasy (Qiagen USA), as described [54]. Splinkerette mapping of transposable elements was performed as described for *D. melanogaster* [55]. Briefly, genomic DNA was digested by BstYI (NEB USA) and the fragments ligated to a Splinkerette element on 5′ and 3′ ends. Fragments containing the insertions were amplified by two cycles of polymerase chain reaction (PCR), using the primers from both ends of the *piggyBac* insertions to the Splinkerette at the ends of the fragments, as designed [60]. The PCR amplicons were sent for Sanger sequencing, and the sequences were aligned against the *An*. *stephensi* SDA500 genome at VEuPathDB, VectorBase (https://vectorbase.org/vectorbase/app/). RNA was extracted as described [54], using Qiagen RNeasy mini kit (Qiagen USA) according to the manufacturer’s instructions. Complimentary (c)DNAs were synthesized using the High-Capacity cDNA Reverse Transcription Kit (ThermoFisher USA) on extracted RNAs. Real-time PCR reactions were carried out on cDNAs using SensiFAST^™^ Real-Time PCR Kits (Bioline USA). The ribosomal protein S7 (*ASTE004816*) was used as a housekeeping control [54].

### Whole genome sequencing

Library preparation and sequencing were conducted at Azenta Life Sciences (South Plainfield, NJ, USA). NEBNext^®^ Ultra^™^ II DNA Library Prep Kit for Illumina was used for library preparation, following the manufacturer’s recommendations. Briefly, the genomic DNA was fragmented by acoustic shearing with a Covaris S220 instrument. Fragmented DNA was cleaned and end-repaired. Adapters were ligated after adenylation of the 3′ ends, followed by enrichment by limited cycle PCR. DNA libraries were quantified using Qubit 4.0 Fluorometer. The DNA libraries were also quantified by real-time PCR (Applied Biosystems, Carlsbad, CA, USA).

The sequencing libraries were clustered onto a 25B flowcell on the Illumina NovaSeq X Plus instrument according to the manufacturer’s instructions. The samples were sequenced using a 2 × 150 paired-end (PE) configuration, targeting ~1 Gb/sample. The control software conducted image analysis and base calling. Raw sequence data (.bcl files) generated by the sequencer were converted into fastq files and de-multiplexed using Illumina’s bcl2fastq 2.20 software. One mismatch was allowed for index sequence identification.

Sequence analysis: To detect transposable element insertions, we used ngs_te_mapper2 (version 1.0.2) [56] with default parameters. Briefly, paired-end Illumina reads were aligned to the *Anopheles stephensi* SDA-500 genome (VectorBase version 68 [57]) using BWA-MEM (version 0.7.17) [58], followed by sorting and indexing with SAMtools (version 1.11) [59]. The pipeline was run using a custom transposable element (TE) library containing the Driver element. Predicted insertions were outputted as BED files annotated with supporting split and discordant reads, insertion frequency, and strand. Genomic context (CDS, exon, intergenic) was determined using bedtools [60] intersect with gene annotations.

Reads can be found under Bioproject PRJNA1281180, and bioinformatics procedures can be found at https://github.com/Franck-Dumetz/Sanaria_transposon.

### Assessment of fecundity

Females were placed individually in plastic *Drosophila* cylinders containing water and sealed with cotton balls 3 days after blood feeding [54]. The mosquitoes were incubated overnight at > 70% humidity and 28 °C. The next day, the mosquitoes were removed from the tubes, and the eggs and larvae were counted in the tubes 1–2 days after egg laying. The larva hatching rate was determined as the percent larvae per the total number of eggs in each tube. Data were analyzed using GraphPad Prism version 10.2.1 for Windows.

## Results

### Generation of *An. stephensi* driver line, expressing rtTA under the vasa promoter

**Fig. 1:**
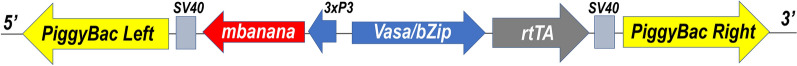
Schematic representation of the driver line construct. The rtTA open reading frame (ORF) was designed to be expressed under the *vasa* or *bZip* promoters. The mBanana is expressed under the *3xP3* promoter. The SV40 3′ untranslated region (UTR) is used as transcription termination for both genes. The cassette was placed between two *piggyBac* transposon elements (yellow arrows)

We constructed a driver line in which the reverse tetracycline transactivator (rtTA, Tet-On) was expressed under the early embryonic promoter, *bZip*. Assessments of rtTA transcript abundance by quantitative real-time (qRT)-PCR revealed very low mRNA levels of rtTA in eggs, ovaries, and adults (data not shown). We therefore replaced the *bZip* promoter with the early embryonic *vasa* promoter. For this *vasa*-rtTA construct, an mBanana fluorescent marker, driven by the *3xP3* promoter was placed in the reverse orientation to the *vasa*-rtTA expression cassette. The construct was placed into a *piggyBac* vector [51] between two transposon elements (Fig. 1) then mixed with a transposase-expressing vector [53] and injected into *An*. *stephensi* eggs at the early embryonic stage. Out of 854 eggs injected, 45 hatched, and 29 (14 males and 15 females) survived to adults. We pooled the adults into four distinct groups: two male (M1 and M2) and two female (F1 and F2) groups, each containing different insertions. Since each group may include multiple insertions, the transformation rate was equal to or greater than 13.8%. Adults were crossed with the opposite sex of wild-type mosquitoes. Larvae of the subsequent generation (G_1_) were screened using a fluorescent microscope, and transgenic larvae expressing mBanana were collected. From the M groups, 114 G_1_ larvae were mBanana-positive, of which 43 males and 39 females survived to adults. A total of 56 mBanana-positive larvae, 27 males and 29 females, were obtained from the F groups, all surviving to adults. To avoid mixing of genotypes, we separated transgenic lines by collecting eggs from 20 individual G_1_ M-group females and 14 individual G_1_ F-group females. The females were set to lay eggs 3 days post-bloodmeal and 10–11 days post-eclosion. After oviposition, RNA was extracted from the G_1_ females and qRT-PCR used to assess rtTA mRNA abundance (data not shown).

### rtTA transcription levels under early embryonic vasa and bZip promoters

We selected four different lines (two from the M group and two from the F group) originating from G_1_ female mosquitoes with the highest abundance of rtTA mRNA. We compared the rtTA mRNA levels of the four mosquito lines (M1f, M2i, F2c, and F2e) to those of three transgenic lines, M1-2, M3-2, and M3-3, we had previously created that express rtTA under the early embryonic *bZip* promoter. The rtTA mRNA level in *bZip*-rtTA M1-2 line was used as a reference. The rtTA mRNA abundance in all three *bZip*-rtTA lines were comparable (Supplementary Fig. S1). In the *vasa*-rtTA lines, mRNA abundance was 1538-fold, 2326-fold, 562-fold, and 380-fold higher in M1f, M2i, F2c, and F2e lines, respectively, than in the reference *bZip*-rtTA line (M1-2) (Supplementary Fig. S1).

**Fig. 2:**
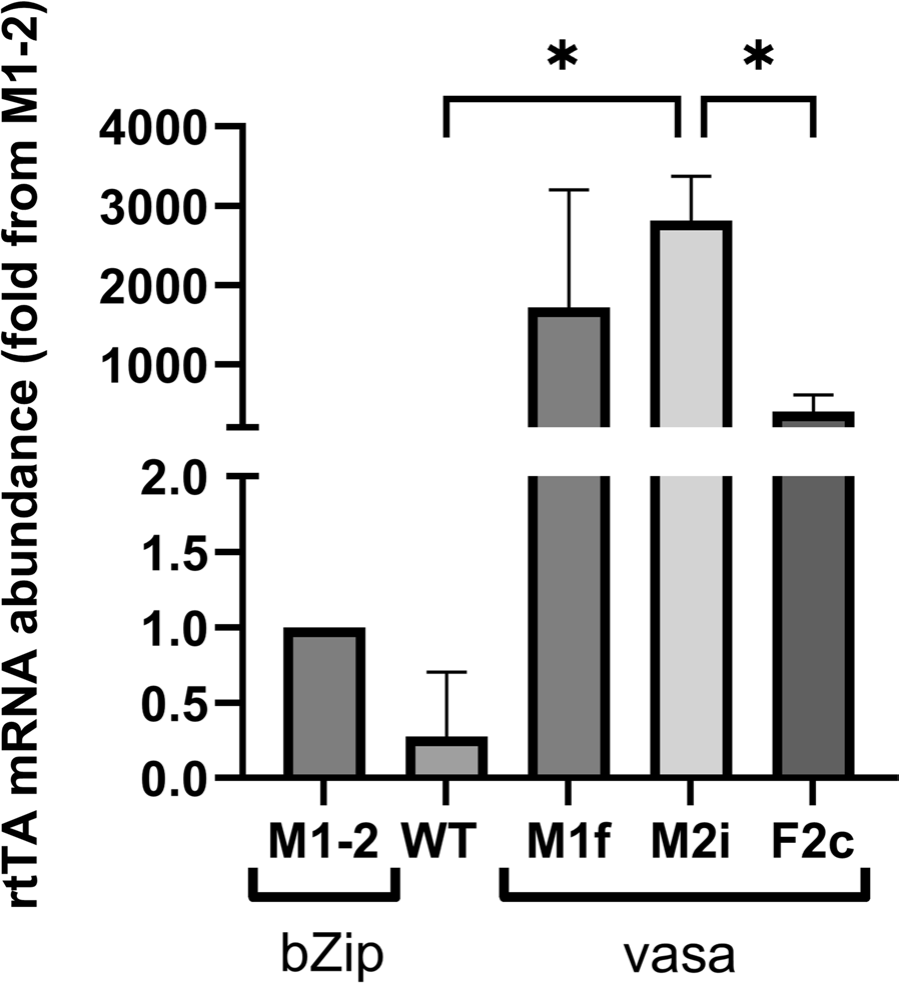
rtTA mRNA abundance in embryos under bZip and vasa early embryonic promoters. Quantitative RT-PCR performed on cDNA from pools of eggs laid by ten females from each group. The results represent the mean mRNA abundances, relative to egg pool from bZip-rtTA M1-2 line ± standard deviation (SD), *n* = 3. The data were analyzed using one-way analysis of variance (ANOVA), ^*^P ≤ 0.05. The ribosomal S7 protein gene was used as the housekeeping gene

Line F2e was lost owing to an insectary failure. We crossed the transgenic *vasa*-rtTA M1f, F2c, and M2i lines to wild-type (WT) through four generations to expand the lines and reduce the number of inserts. Since the *vasa* promoter can drive expression in the early stages of embryonic development [61], we assessed rtTA transcript abundance in embryos 1 h after oviposition (Fig. 2). The mean rtTA mRNA abundance was 1714-fold, 2813-fold, and 405-fold higher than in eggs from the *bZip*-rtTA line M1-2, in the eggs from the M1f, M2i, and M1-2 lines, respectively. As seen in adult mosquitoes, the rtTA mRNA abundance in M2i was higher than in all other lines, and significantly higher compared with F2c embryos (Supplementary Fig. S1 and Fig. 2).

### Mapping the transposon-based insertions of the driver construct and generation of homozygous mosquitoes

From G_6_ onward, the lines were in-crossed to obtain homozygosity of the insertion. We collected genomic (g)DNA at G_7_ and used it to identify the genomic locus of the transposon-based insertion in each line using the Splinkerette PCR protocol (see Methods section). The sequences were aligned against the *An*. *stephensi* genome in VectorBase. In the M1F line, the *vasa* construct was inserted into supercontig KB664888, position 665,462, which is within an exon in the open reading frame (ORF) of an Era G-protein-like (*ASTE010067*) coding sequence. In the F2c line, the insertion was in supercontig KB665088, position 785,341, which is not in any known ORF. In the M2i line, the *vasa* construct was inserted into supercontig KB665343 position, 382,729, which is within the last intron of a putative juvenile hormone diol kinase gene (*ASTE000415*).

**Fig. 3:**
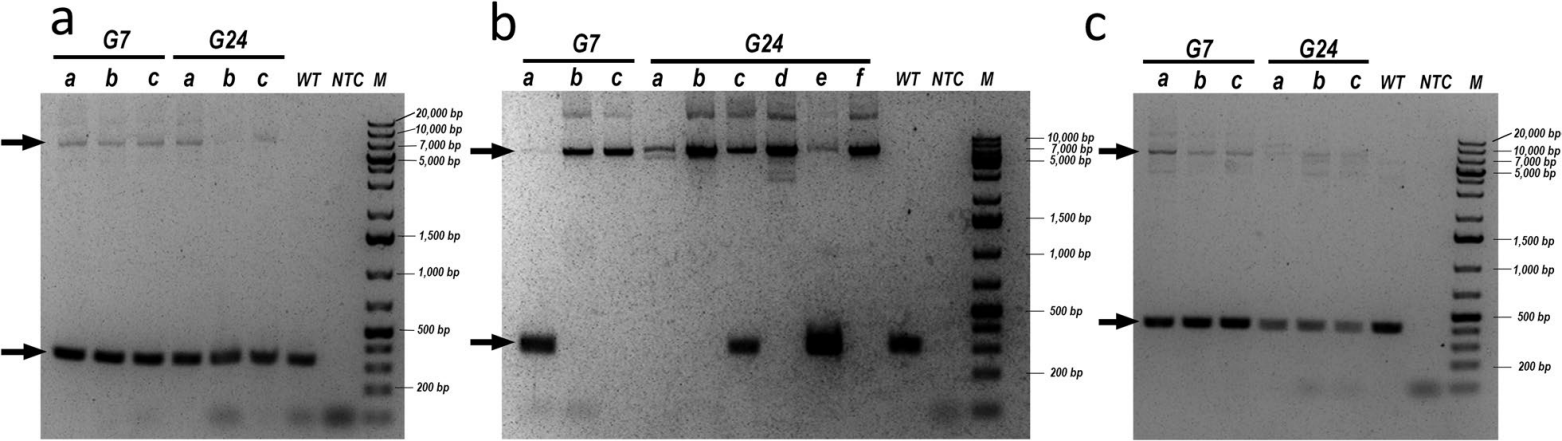
Diagnostic PCR to identify the zygosity status of the insertion in M1f (**a**), M2i (**b**), and F2c (**c**) driver lines. PCR was performed on genomic DNA from three individual mosquitoes (**a**–**c**) from all three lines, collected at G^7^. At G^24^, mosquitoes (**a**–**c**) were collected for M1f and F2c, and six were collected for M2i (**a**–**f**). Genomic DNA from WT *An. stephensi* female mosquitoes was used as a negative control, and water was used as a no template control (NTC). The expected PCR products for the M1F insertion and WT alleles were 7560 and 354 base pairs (bp), respectively. For M2i, the products of the insertion and WT alleles were 7534 and 328, respectively. The expected PCR products for the F2c insertion and WT alleles were 7636 and 430 bp, respectively. Black arrows indicate the position of the PCR products on the gel. Homozygous mosquitoes were found only in M2i

We continued to in-cross the three lines through subsequent generations while monitoring the proportion of mBanana-expressing larvae in each generation as an indication of their progression toward homozygosity. In the M2i line, almost all the larvae were transgenic after G_17_, while in the F2c and M1f lines, only about two thirds of the larvae expressed mBanana. We therefore carefully tracked the proportion of mBanana-positive larvae through generations 24, 25, and 26 (Table 1). All M2i larvae expressed mBanana in generations 24–26. Only 76%, 74%, and 80% of M1f larvae and 77%, 72%, and 78% of F2c larvae expressed mBanana in generations 24, 25, and 26, respectively. On the basis of the positions of the inserts in each line, we designed PCR primers flanking the insertions to determine the zygosity level in each line (Fig. 3). All three M1f- and F2c-tested mosquitoes were hemizygous at G_7_ and continued to be heterozygous at G_24_. Two of three mosquitoes in the M2i line were homozygous at G_7_ (b and c), and four of six mosquitoes (a, b, d, and f) were homozygous at G_24_ (Fig. 3)_._

**Table 1:** Proportion of transgenic mosquitoes expressing the mBanana fluorescent protein after 24, 25, and 26 generations of selection

Line	Generation	mBanana-positive	mBanana-negative	Total	Proportion mBanana-positive (%)
M1f	24	174	54	228	76
	25	194	77	268	74
	26	223	95	318	70
M2i	24	235	0	235	100
	25	264	0	264	100
	26	323	0	323	100
F2c	24	197	60	257	77
	25	173	66	239	72
	26	283	78	361	78

Only the M2i line is shown to be homozygous viable

The presence of hemizygous mosquitoes in the M2i line (Fig. 3B, generation 24, mosquitoes c and e) was unexpected, as all larvae were mBanana-positive (Table 1). This discrepancy likely reflects additional genomic insertions not detected by the Splinkerette protocol. To investigate further, we performed whole genome sequencing (WGS) on genomic DNA from a single M2i female mosquito that was homozygous for the insertion at G_7_ (Fig. 3B, generation 7, mosquito c).

The sequencing yielded 245,363,522 paired-end reads, of which 93.09% (228,407,227) were mapped to the *An. stephensi* genome and 0.29% (701,308) to the transposable element. The sequence data were submitted to the National Center for Biotechnology Information (NCBI) (SRA34189523). Insertions were identified by locating hybrid reads that mapped partially to the *An. stephensi* genome and to either the 5′ or 3′ end of the transposon element. We applied the following filtering criteria: (1) insertions were considered valid if supported by more than ten reads for either 5′ and 3′ reads; (2) if the insert was within 10 bp of the breakpoint; (3) insertions were considered homozygous if > 95% (0.95) of reads at a locus supported the insertion, and hemizygous if the frequency was between 0.3 and 0.7.

Using these criteria, we identified seven unique insertion sites across six scaffolds (Table 2). Two insertions were located on scaffold KB664556, and one on each of scaffolds KB664633, KB664733, KB664943, KB665165, and KB665343. The insertion at KB665343 (position 382725–382726) confirmed the initial finding by the Splinkerette assay. Of the seven insertions, six were homozygous and located in either intergenic regions or introns—including the KB665343 insertion, also validated by PCR (Fig. 3B). The remaining insertion, in scaffold KB665165, was hemizygous and located within an exon of the uncharacterized gene *ASTE001918*.

**Table 2:** Description of the transposon insertions in the homozygous-viable M2i line

Scaffold name	Insertion start	Insertion end	Offset	Frequency	3′ reads	5′ reads	Ref reads	Strand	Gene	Insertion position	Insertion type
KB664556	105925	105932	−7	0.99	67	62	1	+		Intergenic	Homozygous
KB664566	463096	463100	−4	0.97	51	56	2	−		Intergenic	Homozygous
KB664633	1444653	1444659	−6	1	61	36	0	−		Intergenic	Homozygous
KB664733	884951	884961	−10	0.98	76	88	2	−		Intergenic	Homozygous
KB664943	15014	15022	−8	0.95	101	91	5	+		Intergenic	Homozygous
KB665343	382725	382726	1	1	105	103	0	+	ASTE000415	Intron	Homozygous
KB665165	97420	97433	−13	0.58	118	315	226	−	ASTE001918	Exon	Hemizygous

Each row represents a high-confidence insertion supported by split and/or discordant reads. Genomic context was determined by intersecting insertion coordinates with gene annotations. The annotation column summarizes support for each predicted non-reference transposable element (TE) insertion: offset indicates the distance from the reported coordinate to the inferred insertion breakpoint (negative values are upstream); frequency is the estimated proportion of reads supporting the insertion; 3′ reads and 5′ reads represent the number of paired-end reads supporting the junction in 3′ and 5′ of the insert, respectively; and the number of reference reads at that location, as calculated by ngs_te_mapper2

### Assessment of potential insertion-related fitness cost

**Fig. 4:**
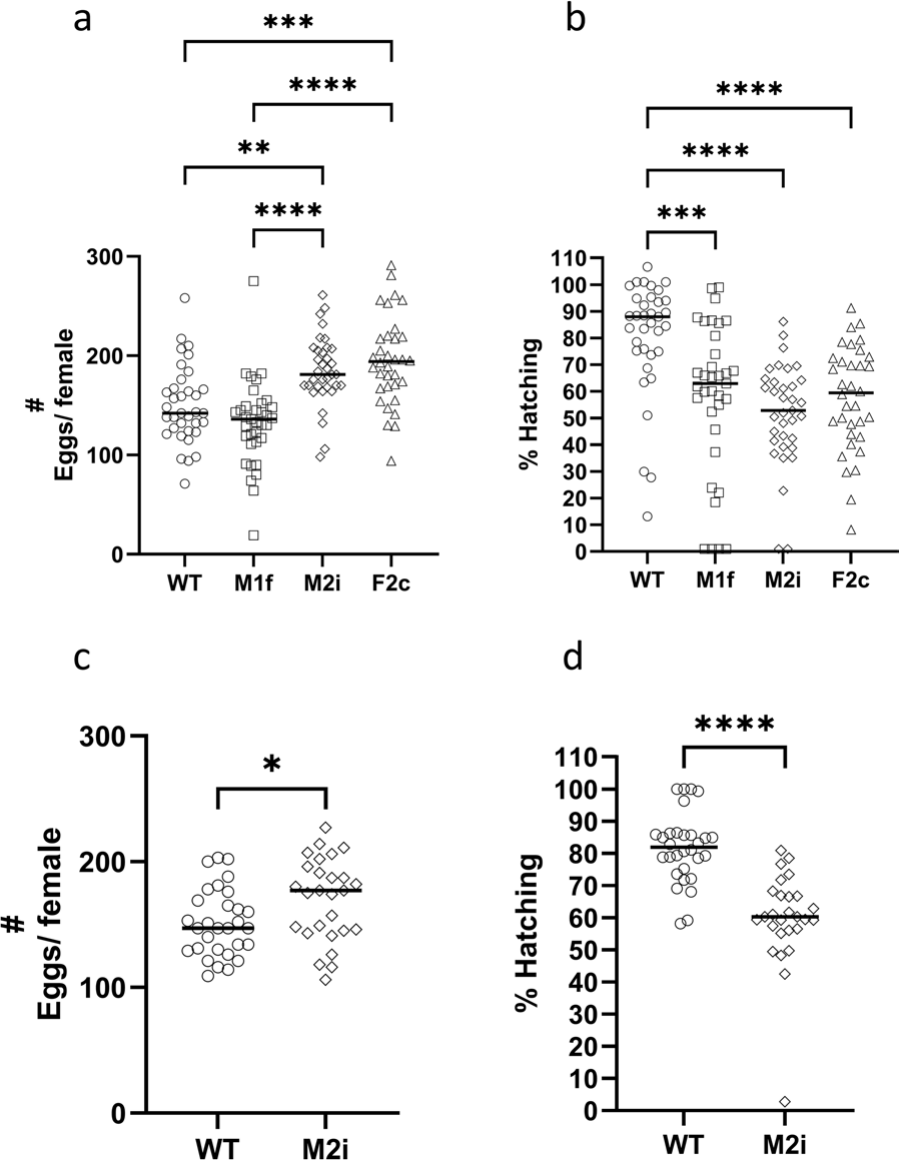
Fecundity in WT and driver line mosquitoes. **a** The number of eggs laid per individual female in *Drosophila* tubes in experiment no. 1. **b** The percentage of eggs hatching in experiment no. 1 was determined by counting the number of larvae in the *Drosophila* tubes 1 day after egg laying. In both panels the results are reported as the geometric means, *n* = 34 for F2c and *n* = 35 for WT, M1f, and M2i lines. The data in both **a** and **b** were analyzed using non-parametric Kruskal Wallis test (^**^ *P* ≤ 0.01, ^***^ *P* ≤ 0.001, and ^****^ *P* ≤ 0.0001). In **b**, some values were 0. Whenever a value was 0, it was arbitrarily assigned a value of 1 to allow calculation of the geometric means. **c** The number of eggs laid per individual female in *Drosophila* tubes in experiment no. 2. **d** The percentage of eggs hatching in experiment no. 2. The data in both **c** and **d** were analyzed using non-parametric Mann–Whitney test (^*^ *P* ≤ 0.05, ^****^ *P* ≤ 0.0001)

To assess the effect of the rtTA expression on fitness, we compared the fecundity in all three lines with that of WT *An*. *stephensi*. From each line, eggs from up to 100 individual females were counted and the proportion of eggs hatching determined. From the WT line, 83% laid eggs. Likewise, 86% and 84% of the females laid eggs in the M2i and F2c groups, respectively. Only 59 out of 86 (68%) M1f females laid eggs. The geometric mean (GM) number of eggs laid per individual female in the WT line was 145, (95% confidence interval (CI) 133–159). In the M1f line, the number of eggs per female was slightly but not significantly lower (GM = 123, 95% CI 106–143), while the numbers of eggs laid by M2i (GM = 181, 95% CI 169–195) and F2c (GM = 190, 95% CI 175–207) females were significantly higher than those of WT and the M1f line (Fig. 4a). The GM of the percentage larvae hatching in WT was 76% (95% CI 65–88). Hatching rates were significantly lower in all three driver lines, 38% (95% CI 45–63) in M1f, 42% (95% CI 30–59) in M2i, and 53% (95% CI 45–62) in F2c (Fig. 4b). In the following generation, the number of eggs laid per M2i female (GM = 167, 95% CI 154–181) was again higher than WT females (GM = 149, 95% CI 139–159) (Fig. 4c), and the hatching rate in M2i (GM = 55%, 95% CI 44–70) was significantly lower compared with that of WT (GM = 81, 95% CI 77–85) (Fig. 4d), confirming the previous observation (Fig. 4a, b).

## Discussion

Our long-term goal is to establish a system for conditionally expressing foreign genes in *An*. *stephensi*. Our medium-term goal is to use this system to conditionally express a lethal gene in male mosquitoes to increase the efficiency of our manufacturing process. In the present study, we created the driver line for the conditional expression that can be crossed with the effector line to generate mosquitoes with the desired phenotype.

We evaluated two promoters for driving expression of rtTA. For unknown reasons, transcription of rtTA was extremely low under *bZip*. This finding is consistent with a previous study in *Ae. aegypti*, which showed that while the *bZip* promoter effectively drove strong transcription of the mNeonGreen fluorescent (mNG) marker, it failed to initiate transcription of tTA [62]. In contrast, the *vasa* promoter facilitated high levels of rtTA expression. The *vasa* promoter has been shown previously to drive endogenous *vasa* gene expression as well as exogenous eGFP expression in ovaries, testis, and 1–2 h post-oviposition embryos of *An. gambiae* [61].

We were unable to take the M1f and F2c lines to homozygosity. In contrast, homozygous mosquitoes were successfully produced in M2i line, as early as G_7_. The expression of rtTA mRNA in M2i was higher than in M1f and F2c, likely due to multiple insertions in the M2i line, where in six out of seven insertions the rtTA encoding gene was present on both chromosomes rather than just one. In M1f and F2c, the frequency of mBanana-positive larvae and the PCR results suggested only a single insertion in a hemizygous state. Therefore, it appears that the inability to reach homozygosity in M1f and F2c was not attributable to any rtTA expression-related toxicity but rather to the genomic locus of the insertions.

In the M1f line, the rtTA was inserted within an exon of a coding gene, Era G-protein-like (*ASTE010067*), a member of a family of G-proteins associated with signal transduction and involved in many aspects of the life cycles in many organisms. One essential, well-known function of G-proteins in insects, including anophelines, is in the sensing of environmental signals through odorant-binding proteins and the response of the insects to those signals [63, 64]. It is therefore highly likely that *ASTE010067* is an essential gene, and the mosquitoes cannot tolerate disruption of both of its copies. In F2c, the insertion was at the 3′ end of supercontig KB665088, in a non-coding region, which may be crucial for mosquito survival.

Seven distinct insertions were identified in the mosquito from the M2i line, six of which were homozygous and located within intergenic or intronic regions. One insertion on supercontig KB665343 was found within an intron of a putative juvenile hormone diol kinase gene (*ASTE000415*). In the moth *Heortia vitessoides*, this gene is involved in juvenile hormone recycling and is essential for development and survival [65]. The fact that this insertion reached homozygosity suggests it did not disrupt the gene’s expression or function. In contrast, another insertion occurred within an exon of the uncharacterized gene *ASTE001918* and was present in a hemizygous state, indicating that complete disruption of this gene may be lethal and that it is likely essential for mosquito viability. As the sequencing was carried out in G_7_, we do not know if this specific insertion had been sustained in later generations. If the insertion persists, we will eliminate it in future generations by PCR screening and selectively removing mosquitoes that carry it.

The homozygous-viable line, M2i, produced significantly more eggs (24% and 12%) than the WT mosquitoes in two experiments (Fig. 4). However, M2i had a significantly lower (45% and 33%) rate of hatching (Fig. 4), reducing the overall production of larvae by about 21%. Since the driver line will next be crossed with an effector line, it remains to be seen whether this reduced hatching rate will impact production of adults in the final product. Expression of tTA has been associated with fitness costs in insects. Overexpression in *D. melanogaster* had a toxic effect on early development in heterozygous progeny due to interference in ubiquitination and protein proteolysis as well as transcriptional squelching [40]. Thus, it was used later as a lethal gene in RIDL systems in *Ae. aegypti* and *D. melanogaster* [39, 45]. The lethal effect of tetracycline-repressible transcriptional activator variant (tTAV), a codon-optimized version of tTA, on transcription in *D. melanogaster* is dependent on the site of the genomic integration. All insertions have negative effects on early development, but each insertion triggers a different transcriptional profile leading to different phenotypes [39]. On the basis of these observations, Oxitec developed transgenic mosquitoes in which females are being killed in the field by overexpressing the tTA in a positive feedback loop [45, 66, 67]. In these mosquitoes, only minimal levels of tTA are being produced when tetracycline is present in the larval diet, but when these mosquitoes are released in the field with no tetracycline, the tTA binds to TetO allowing overexpression of the downstream tTA in females, and mortality of subsequent mosquito generations at the early larval stage. Release of these mosquitoes in field studies in the USA and Brazil led to reductions of up to 96% of the mosquito populations [46, 49].

## Conclusions

We successfully completed the first step in creating *An*. *stephensi* with a male-lethal allele controlled by a conditional gene expression system. We produced the *An*. *stephensi* homozygous-viable driver line, M2i, that expresses rtTA under the early embryonic *vasa* promoter. Egg production by M2i was ~18% greater than by WT mosquitoes, and the hatching rate of eggs to larvae ~39% lower than in WT mosquitoes. This overall 21% decrease in fecundity is acceptable for moving to the next step, which will be to cross the M2i driver line with an effector line carrying a male-lethal gene. In the final product, the rtTA transcription factor will bind to the tetracycline responsive element (TRE) in the presence of doxycycline, driving lethal gene expression only in males. Success in step two will provide the foundation for crossing the driver line described herein with other lines to conditionally express any target gene in *An*. *stephensi*.

## Supplementary Information

**Additional file 1: Figure S1.** rtTA mRNA abundance in adult females under *bZip* and *vasa* early embryonic promoters. Quantitative RT-PCR done on cDNAs produced from RNAs, extracted from individual female adult mosquitoes expressing rtTA using the *bZip* or *vasa* promoters, following egg laying. The abundances are relative to a female mosquito from *bZip*-rtTA M1-2 line. The ribosomal S7 protein gene was used as the house keeping gene.

## Data Availability

All data generated or analyzed during this study are included in this published article.
